# Urban Environment and Health: A Cross-Sectional Study of the Influence of Environmental Quality and Physical Activity on Blood Pressure

**DOI:** 10.3390/ijerph18116126

**Published:** 2021-06-06

**Authors:** Regina Grazuleviciene, Sandra Andrusaityte, Audrius Dėdelė, Tomas Grazulevicius, Leonas Valius, Aurimas Rapalavicius, Violeta Kapustinskiene, Inga Bendokiene

**Affiliations:** 1 Department of Environmental Science, Vytautas Magnus University, 44248 Kaunas, Lithuania; sandra.andrusaityte@vdu.lt (S.A.); audrius.dedele@vdu.lt (A.D.); t.grazulevicius@gmail.com (T.G.); 2 Department of Family Medicine, Lithuanian University of Health Sciences, Eivenių str. 2, 50161 Kaunas, Lithuania; Leonas.Valius@lsmuni.lt (L.V.); Aurimas.Rapalavicius@lsmuni.lt (A.R.); Violeta.Kapustinskiene@lsmuni.lt (V.K.); 3 Division of City Planning and Architecture, Kaunas City Municipality, Laisvės al. 96, 44251 Kaunas, Lithuania; inga.bendokiene@kaunas.lt

**Keywords:** urban infrastructure, environmental quality, citizen science, neighborhood perceptions, hypertension, physical activity

## Abstract

Few studies have examined the relation between urban built environment and the prevalence of hypertension. This cross-sectional study aimed at assessing the relationship between the environmental quality, physical activity, and stress on hypertension among citizens of Kaunas city, Lithuania. We conducted a survey of 1086 citizens residing in 11 districts to determine their perceptions of environmental quality, health behavior, and health indices. The independent variables included residential traffic flows, access to public transportation and green spaces. Dependent variables included physician-diagnosed hypertension, systolic and diastolic blood pressure, and stress level. We used multivariable logistic regression to assess the associations as odds ratios (OR). The environmental factors beneficially associated with meeting the physical activity recommendations were opportunities for walking to reach the city’s green spaces and available relaxation areas. Residents of high noise level districts aged 45–64 years had a significantly higher OR of stress and a higher prevalence of hypertension when age, sex, education status, family status, and smoking were accounted for. However, meeting the physical activity recommendations had a beneficial effect on the risk of hypertension. This study provided evidence that improvement of the district-level built environment supporting citizens’ physical activity might reduce the risk of hypertension.

## 1. Introduction

Over the past decade, an increasing number of studies have reported on the impact of the urban environment on citizens’ health. The degree of urbanization and exposure to various environmental stressors and traffic-related air pollutants and noise have been associated with a higher risk of health problems [1,2,3,4]. The urban built environment influences major determinants of health, such as neighborhood traffic exposure, physical activity, chronic stress, and social cohesion. The neighborhood in which people live has a varying-level impact on physical, mental, and general health and well-being [5,6,7]. Some study findings have provided evidence that neighborhood environment has an independent influence on cardiovascular disease and hypertension—the second leading cause of chronic diseases worldwide [8,9,10]. Hypertension has been identified as an environment-related disease, and environmental stressors, such as noise, air pollutants or psychosocial factors may have an impact on the increase in blood pressure [11,12]. Chronic stress has been suggested as a risk factor for hypertension and cardiovascular disease. Some studies suggest that oxidative stress takes part in the development of hypertension [13]. Thus, environmental stressors that produce excessive sympathetic nervous system activity and alterations in the activity of the neuroendocrine and the immune systems through stress reactions take part in the pathogenesis of hypertension and cardiovascular disease [7,14,15].

Recent research findings suggest that the quality of the neighborhood environment may play an important role in reducing the risk of chronic non-communicable diseases [16]. There is some evidence that green space in the neighborhood is related to citizens’ physical activity and the level of stress [16,17,18]. Citizens living in more urbanized areas spend less time on green space visits and physical activity [19,20], while people living in neighborhoods with higher surrounding green spaces more often meet physical activity recommendations, feel less stressed, and experience higher social cohesion [4,21,22]. Recent epidemiological studies have reported different levels of associations between exposure to green spaces and hypertension [16,23]. To date, the pathways of greenspace-related health benefits are unclear [24,25,26]. However, some study findings suggest that physical activity in green spaces may have a positive impact on cardiovascular health by reducing stress level [27,28,29].

Epidemiological findings demonstrate that the major determinants of health may be modifiable through environmental policy changes or health behavior changes influencing citizens’ blood pressure. Most of the studies that demonstrated beneficial effects of physical activity on reductions in blood pressure were randomized controlled trials and comprised hypertensive adults [30,31]. Investigation of the effects of isometric exercise on blood pressure in healthy adults showed the potential to produce clinically meaningful blood pressure reductions [32]. However, recent clinical findings revealed that response to aerobic exercise differ from response to isometric exercise [33]. Several epidemiological studies, that analyzed the association between the recommended levels of aerobic physical activity and blood pressure in middle-aged adults showed different proportion of participants meeting the guidelines of a minimum of 30 min moderate-intensity activity per day [34,35] and an inconsistent anti-hypertensive response to physical activity [36,37,38,39]. Most studies have been limited by focusing on middle-aged men of European countries. Differences in environmental factors, physical activity levels and genetic factors may produce a variability in the blood pressure response to physical activity at the population level [40]. A recent systematic scoping review of the neighborhood social and built environment and physical activity highlighted the need for further studies in which environmental factors would be key influencers of physical activity [41,42]. There is some indication that the investigation of the context-specific built environment associations and improving neighborhood walkability and the quality of parks may have a positive impact on the policy change process and may increase physical activity [43,44,45]. To date, even though some findings present various levels of associations between the urban environment and physical activity, the evidence concerning the effects of the recommended levels of aerobic physical activity on blood pressure in different environment remains inconclusive.

To our knowledge, this is the first study to investigate the influence of the perceived quality of urban built environment and recommended physical activity on blood pressure and stress levels among 45–64-year-old citizens of an Eastern European country. This study is one of the first to explore the associations between satisfaction with the objectively measured and the subjectively measured (using a questionnaire) quality of the built neighborhood, physical activity, and self-reported health indices. The study was initiated as the Kaunas study of the Horizon 2020 proposal Citizen Science for Urban Environment and Health [46]. The previously published findings showed that the poor quality of the neighborhood and individual-level stressors were those very factors that influenced a higher prevalence of health problems at the district level in 18–75-year-old citizens [11]. Differently from earlier studies using a small proportion of the group of 45–64-year-old participants, the present study used data collected from 11 Kaunas neighborhoods, which have large populations in the age group of 45–64 years and are heterogeneous in environmental settings, and analyzed the relationships between self-reported noise levels, physical activity, stress, and the risk of hypertension in the group of 45–64-year-old individuals.

This study had three objectives: (1) to examine the impact of the quality of the urban built neighborhood on citizens’ physical activity; (2) to estimate the relationship between the quality of the built environment, physical activity, and health indices in 45–64-year-old participants; and (3) to examine if physical activity moderates the relation between environmental quality indicators and hypertension.

The complex research of the urban built neighborhood in relation to physical activity, stress, and hypertension has the potential of producing evidence on health effects of modifiable environment-related and behavioral factors for interventions to improve all citizens’ cardiovascular health and well-being.

## 2. Materials and Methods

### 2.1. Study Design

This citizens’ collaborative study was conducted during 2019–2021 and consisted of two stages of the participants’ enrolment. The survey targeted 1086 participants. During the first stage of the engagement, 580 18–75-year-old participants were enrolled. During the second stage, 506 45–64-year-old participants randomly selected from 11 districts of Kaunas city, Lithuania, were enrolled. The area of Kaunas city is 15,700 hectares, 8329 hectares of which are covered by greenery (parks, groves, gardens, natural reserves, and agricultural areas). All Kaunas city parks are open to the public, are located amidst residential homes or establishments and near public transport lines, and offer some recreation opportunities (e.g., walking, jogging, rollerblading, physical training, or resting on the bench). The city green spaces measured with the NDVI included parks, groves, green roofs, streams, and community gardens. The mean NDVI in Kaunas city was 0.571. Such greenness level shows an area containing a dense vegetation canopy. In Kaunas, about 95% of the citizens see green spaces from home and have a convenient access to a city park by walking from homes for 10 min or less. Such measurements were suggested as a European Commission environmental quality indicator of green urban areas [47].

A detailed description of the methods of the participants’ enrolment as well as the description of the study have been provided previously [11,48]. The participants together with the scientists were involved in identifying the relevant questions, contributed to data collection, identified environmental problems in the district and health concerns, and participated in the discussion on the study questionnaire. During the surveys, the participants filled in a formalized questionnaire that had closed-ended and open-ended questions. All participants gave their informed consent for inclusion before entering the study. The study was conducted in accordance with the Declaration of Helsinki [49]. The study protocol, the questionnaire, and the consent procedure were approved by the Kaunas Regional Committee for Biomedical Research Ethics (BE-2-51. 2019-06-10).

Using an environmental epidemiological approach, we conducted a cross-sectional study seeking to assess the relationship between the environmental quality of the residential area, physical activity, stress level, and the prevalence of hypertension. We conducted an analysis of our survey participants’ personal characteristics and addresses linked to geographic information systems (GIS) data describing their neighborhoods. We used geospatial analysis and adjusted multi-level models to assess the association between the objectively measured and the subjectively measured (using a questionnaire) quality of the built neighborhood on meeting the physical activity recommendations, stress level, and the prevalence of hypertension by controlling for the influence of possible confounding variables.

### 2.2. Measurements

The transport network of Kaunas city has an annular-radial structure. In this study, exposure to major traffic flows was estimated by the GIS. Residential addresses were used to estimate traffic-related exposure indices. The participants residing on a street with above 10,000 cars/day were classified as exposed to heavy traffic emissions in their place of residence. We collected personal data on baseline demographic, lifestyle, and health history characteristics and subjective rating on residential district exposures by using standardized questionnaires. Information collected through questionnaires covered socio-demographic and health-related data as follows: age, sex, family status, self-reported health, smoking, physical activity, socio-economic situation (SES), and residence history. We measured the participants’ perception of the environmental quality by asking the participants to rate answers to questions about their neighborhood and built environment. The participants’ perception of the characteristics of the district’s built environment was assessed using questions on the infrastructure in the residence neighborhood: public transport, pathways and cycling routes, walking distances to the city’s green spaces or parks, and areas adapted for exercise and relaxation. The questionnaire included questions on opportunities for societal relationships and social well-being: public spaces to meet people, social cohesion and inclusion among different groups, the feeling of safety in the place of residence, and stress level. In addition, we asked questions on environmental quality issues such as problems caused by air pollution and noise in the place of residence as well as on the average time per day spent outdoors when engaged in physical activity such as fast walking, biking, or gardening. The responses were scored using a seven-point Likert rating scale ranging from 1 (strongly disagree) to 7 (strongly agree) to measure mean environmental perceptions. Scores above mean indicated higher quality and better neighborhood conditions.

Hypertension was defined using the criteria set by the European Society of Cardiology and European Society of Hypertension [50]—the presence of physician-diagnosed hypertension, the reported use of antihypertensive medication, and/or systolic blood pressure of 140 mmHg or higher and/or diastolic blood pressure of 90 mmHg or higher. The accuracy and consistency of the study participants’ reporting of physician-diagnosed hypertension was based on the comparison of the data with responses on blood pressure readings and the prevalence of self-reported physician-diagnosed hypertension, and the comparison with the measurements of a representative sample of the inhabitants of Kaunas city [51].

We collected information on physical activity during the previous week by obtaining the participants’ feedback about the mean time per day spent outdoors for fast walking, biking, or gardening. The consistency of answers on time spent outdoors was based on comparisons with time spent in a park, and with the responses of a random sample of Kaunas citizens [52]. The participants were then classified into two groups according to the Public Health Guidelines for Physical Physical Activity [53], i.e., at least 150 min/week of moderate-intensity physical activity outdoors (the recommended duration), or fewer min/week spent outdoors (low physical activity).

**Figure 1:**
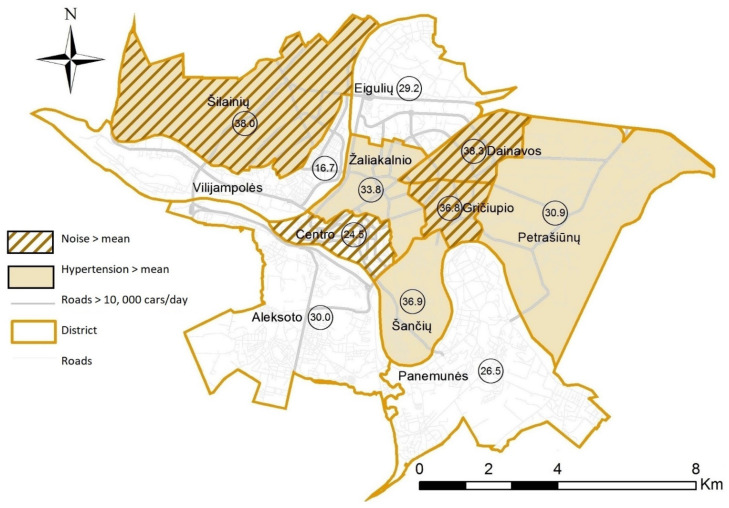
Spatial distribution of exposure to noise (by mean) and the unadjusted prevalence of hypertension (%) at the Kaunas district level among 45–64-year-old participants.

We geocoded individual residential addresses provided by the participants to create variables with the residence district/community categories. Figure 1 presents GIS-visualized 45–64-year-old participants’ perception of noise exposure and spatial distribution of the unadjusted prevalence of hypertension at the Kaunas district level, using the ArcGIS 10.4 mapping and analytics platform (Esri, Redlands, CA, USA).

### 2.3. Statistical Analysis

In the statistical analysis, we dichotomized personal data and used mean values of environmental perception scores as cut points for an easier interpretation of logistic regression estimates. The frequency distributions of all participant characteristics were tabulated for the analytic sample. First, we used the chi-squared test to compare the values and the frequencies of the participants’ baseline characteristics and mean ratings of the perceptions of neighborhood quality and social well-being by the physical activity level. Quantitative variables were reported as mean values and standard deviations. Statistical significance was set at *p*-value <0.05. We used Fisher’s exact tests to compare the qualitative characteristics between the groups. Second, we applied multivariable logistic regression models to estimate the relationship between the quality of the built environment and physical activity and hypertension, diastolic blood pressure, and stress level in 45–64-year-old participants. The relationship between the variables was estimated as odds ratios (OR) and their 95% confidence intervals (CI), controlling for possible confounding variables such as sex, education level, age, smoking status, and income. In the multivariable logistic regression models, we applied higher than 0.05 *p*-value thresholds (such as <0.2) for the inclusion of predictor variables from bivariate statistics in order to prevent the exclusion of relevant factors [54]. For this reason, we also retained the variables that changed the adjusted odds ratios (aOR) by 10% or more for inclusion in the multivariable logistic regression analysis. Statistical analyses were performed using SPSS version 25.0 package (IBM Corporation, New York, NY, USA).

## 3. Results

### 3.1. Participants’ Characteristics by Physical Activity Level

To identify personal characteristics associated with the physical activity level, we performed a total sample analysis by low (less than 150 min/week of moderate intensity activity) and recommended (at least 150 min/week of moderate intensity activity) levels of physical activity (Table 1, Table 2 and Table 3). We sought to identify the determinants or risk factors for health outcomes. The generated *p*-values were not adjusted for multiple testing and should be interpreted exploratorily only.

The participants of this study were 1086 citizens; most of them were of working age, about 33.1% of the participants were 18–44 years of age, 61.5% were 45–64 years of age, and 5.3% were aged 65 years or older. As many as 53.1% of all the participants had university degrees. As shown in Table 1, about 28.5% of the participants had been exposed to heavy traffic emissions (above 10,000 cars/day) in their place of residence for about 17 years.

Geographic information is an important aspect of the visualization of the relationships between the built environment and health outcomes. We used a mapping database of GIS, which provided insights about the relationship between proximity to streets with heavy traffic and hypertension outcomes. We explored spatial patterning in noise perceptions (by the <mean or >mean score) and the prevalence of hypertension (in percent) at the district level, and a map of Kaunas city was created (Figure 1). The transport network of Kaunas city has an annular-radial structure. The mean prevalence of hypertension in 45–64-year-old participants was estimated according to the self-reported data of the study participants, and it was found to be 30.2%. The prevalence of hypertension in various districts ranged from 16.7% to 38.3%. These data were not adjusted for possible confounding variables for hypertension, such as age, sex, education level, smoking, or income, and environmental stressors. Therefore, unequal distribution of these variables in the districts might have had an impact on the prevalence of hypertension. For this reason, univariate analysis without controlling for possible confounding variables would not be able to explain what impact high noise levels had on the prevalence of hypertension in 45–64-year-old citizens in Kaunas districts.

Unadjusted factor analysis by levels of physical activity (Table 1) showed non-significant differences in physical activity between the three age groups of the participants, between participants of different residence districts and exposure to traffic flows, or between participants with different SES (*p* > 0.05). However, significant differences in health characteristics were found between the participants of different physical activity groups (Table 2). Among 45–64-year-old participants, those with a low physical activity level suffered from hypertension more often than participants with the recommended physical activity level (31.9% and 21.8%, respectively, *p* = 0.041), had a higher mean diastolic blood pressure (*p* = 0.017), and more often reported higher stress level (*p* = 0.007). These results of the unadjusted factor analysis showed links between the self-reported health indices and recommended physical activity only in 45–64-year-old participants, indicating that low physical activity might have affected health indices. Since we did not find any similar associations in other age groups, our further analysis comprised only the group of 45–64-year-old participants.

### 3.2. Participants’ Perceptions of Neighbourhood Quality Characteristics by Physical Activity Level

In order to identify the impact of the quality of the urban built neighborhood environment on citizens’ physical activity, we examined whether physical activity depended on the participants’ perceptions of the neighborhood quality and social well-being (Table 3). Higher scores indicated better neighborhood quality and social well-being, and less stress.

The participants of both groups (low physical activity and recommended physical activity) similarly (*p* > 0.05) highly rated the public transport in the district, indicating that it met their needs (5.15 and 5.44, respectively), were satisfied with pathways and cycling routes, and similarly acknowledged the possibility for exercise and relaxation in the residential area. There were no significant differences between the groups concerning the evaluation of problems caused by air pollution and noise in the place of residence, in the feeling of safety in the place of residence, social cohesion, or involvement in decision-making. However, the participants who reached the recommended level of physical activity more often than those with low physical activity levels acknowledged the good opportunities for walking to reach the city’s green spaces or parks (5.67 and 5.26, respectively), regularly visit the natural environment (5.46 and 4.66, respectively), and public spaces and rooms to meet people available in the residential area (4.49 and 3.99, respectively). The question “During the last 6 months, have you felt stress, tension, or anxiety?” received significantly higher scores (less stress) in the participants who reached the recommended physical activity level (4.08 and 4.68, *p* = 0.002). These results show that the quality of the built neighborhood environment did have an impact on physical activity levels and that the participants who reached the recommended physical activity level felt stress less frequently than those with low physical activity levels did.

### 3.3. The Relationships between Perception of Environmental Quality, Physical Activity, and the Risk of Health Issues

We used three multivariable logistic regression models to examine the associations between neighborhood quality and physical activity, and hypertension, diastolic blood pressure, and stress, considering both individual-level and neighborhood-level variables.

Table 4 presents odds ratios (OR) and 95% confidence intervals (CI) from the multivariable logistic regression models examining covariates and associations. In this analysis, the referent group was the mean score or above the mean. The first model included hypertension and scoring of the environment-related perceptions, and physical activity. The second one included diastolic blood pressure, and the third model included stress. The data of all the models were adjusted for sex, education level, age, smoking status, and income. The first model showed significant relationships between regular visits to green spaces, available relaxation area, noise problems, and hypertension. Rare visits (<mean) to green spaces were associated with increased adjusted odds ratios of hypertension (OR 1.80 (95% CI 1.27–2.55)), unavailability of a relaxation area was associated with odds ratios of hypertension 1.69 (1.20–2.36), while noise problems increased the risk of hypertension by 47%, (OR 1.47 (95% CI 1.03–2.08). The feeling of safety in the place of residence was not significantly associated with hypertension. Less physically active participants had significantly higher odds ratios for hypertension (OR 1.65 (95% CI 1.01–2.67)) than participants with the recommended physical activity level did. Diastolic blood pressure of 90 mmHg or higher was associated with rare visits to green spaces (OR 2.09 (95% CI 1.39–3.13)). Poorer quality of the built and social environment significantly increased odds ratios for stress. Low physically active participants also had significantly higher odds ratios for stress (OR 1.79 (95% CI 1.18–2.72)) than participants with the recommended level of physical activity did. These findings show that noise in the place of residence that impaired sleep and/or work at home and low physical activity were variables associated with stress and hypertension.

To explore the role of physical activity as a factor modifying the effects of noise level on hypertension in 45–64-year-old participants, we conducted a stratified multivariable logistic regression analysis (Table 5). During this analysis, we controlled for the influence of possible confounding variables and analyzed if the strength of the associations between noise level and hypertension differs depending on the value of third variable i.e., physical activity.

In this analysis, the referent group was comprised of participants who had low noise levels in the place of residence and achieved the recommended physical activity level. Among the participants of this group, the prevalence of hypertension was 19.0%, while among the participants with a high noise level in the place of residence, the prevalence of hypertension was 29.0%. The multivariable logistic regression model showed that, irrespective of whether the recommended physical activity level was achieved, a high noise level tended to increase odds ratios for hypertension by about 93% (OR 1.93, 95% CI 0.73–5.13). Similarly, non-significant results were among the participants with a low noise level and low physical activity. However, the combination of a high noise level and low physical activity was associated with a significant increase in the risk of hypertension in the unadjusted (OR 2.49, 95% CI 1.32–4.71) and the adjusted (OR 2.25, 95% CI 1.28–4.69) models. These results revealed that the recommended physical activity modified the effects of noise on hypertension in 45–64-year-old participants, and both a high noise level and a low physical activity level contributed to the risk of hypertension in this group.

## 4. Discussion

This citizen science research presented evidence about the links between the quality of the urban built neighborhood environment and citizens’ physical activity, stress level, and self-reported hypertension. To our knowledge, there are only a few studies on the associations between the neighborhood characteristics and residents’ perceptions of their neighborhood quality and links with hypertension. However, this is the first citizen science study to explore the role of recommended physical activity as a modifying factor for the effects of noise on hypertension in 45–64-year-old participants in an Eastern European country. The obtained findings showed that the quality of the built neighborhood environment had an impact on the physical activity level and that the participants who reached the recommended physical activity level felt stress less frequently than those with low physical activity levels did. Our study showed that 45–64-year-old participants with a low physical activity level more often suffered from hypertension, had a higher mean diastolic blood pressure, and more often reported a higher stress level than participants who achieved the recommended physical activity level did. The results of the multivariable analysis showed that the self-reported health indices in 45–64-year-old participants might have been the outcome of a combined effect of poor environmental quality and low physical activity. Our results demonstrated that both perceived high noise levels and low physical activity contributed to the risk of hypertension in 45–64-year-old participants.

The findings of this study are consistent with the results of an international study that reported that neighborhoods with high walkability may have a positive effect on the risk of hypertension at the community level [55]. Our results showed that good opportunities for walking to reach the city’s green spaces or parks and to regularly visit the natural environment in neighborhoods had a strong influence on reaching the recommended physical activity level and on reduction in the stress level. Among the environmental factors, the perception of safety in the district had the highest impact on the stress level (adjusted OR 2.16, 95% CI 1.58–2.95). The neighborhood quality items, and the reached recommended physical activity level had a beneficial effect on the stress level and the risk of hypertension. Specifically, in support of our hypothesis that physical activity acts as a modifying factor for the effects of noise on hypertension, we have shown that low physical activity had a significant impact on an increase in the risk of hypertension in high-noise neighborhoods.

Beyond the positive association between the satisfaction with the neighborhood quality and meeting the physical activity recommendations, we also found a beneficial impact on self-reported health characteristics, such as lower diastolic blood pressure, the prevalence of hypertension, and stress in 45–64-year-old participants. The new findings from the current study point to the important role of recommended physical activity in moderating the effect of noise and chronic stress on hypertension. The findings of this study are partially consistent with the scientific evidence that both physical activity and contact with urban green spaces have the potential to contribute positively to citizens’ health [29,56,57], and that individual-level characteristics may independently contribute to health outcomes such as blood pressure [28,58]. Our findings are in line with the previously reported data indicating that the neighborhood environment may influence blood pressure through physical activity and experience higher social cohesion in the neighborhood, suggesting biological and psychosocial pathways linking exposure to green spaces with hypertension [16,24,25,26].

In this study, the level of physical activity did not depend on the 18–75-year-old participants’ socio-demographic characteristics. High levels of satisfaction with the participants’ neighborhood infrastructure created motivation for physical activity. However, the physical activity was low among participants of all age groups, and most of the study participants did not reach the recommended physical activity level. Only 11.9% of the participants in the age group of 18–45 years and 16.5% of the participants in the age group of 45–64 years reached the recommended physical activity level. Self-reported health characteristics of the 45–64-year-old participants with low physical activity levels were significantly poorer compared to those of the participants who achieved the recommended physical activity level: the participants of the former group suffered from a higher mean diastolic blood pressure, a higher stress level, and a higher prevalence of hypertension. We did not find such consistent pattern in the age groups of 18–44 years or 65 or more years. Such results may partially explain inconsistent associations between environmental exposures and health outcomes reported in epidemiological studies because these relationships are often affected by poorly controlled confounding factors [59].

Our findings are in line with the previously reported data indicating that blood pressure may be related to neighborhoods through multiple mechanisms and is associated with modifiable and non-modifiable risk factors such as behavioral, social, and environmental risk factors that might produce stress and contribute to hypertension [56,60]. A decrease in the hemostatic markers of inflammation has been suggested as a mediating mechanism for the beneficial effect of physical activity on the risk for cardiovascular disease [61]. Our observed self-reported lower stress levels and changes in the risk of hypertension among physically active participants indirectly support this hypothesis. The modification of the noise-associated effect on the risk of hypertension was observed among participants who reached the recommended physical activity level.

### Strengths and Limitations of the Study

The strengths of this citizen science study, in relation to other studies, include an environmental epidemiological approach and standardized methods for the assessment of associations. These measures helped us to gain new knowledge on the impact of the environmental quality on recommended physical activity and self-reported health issues. Using GIS, we were able to estimate every participant’s address and to analyze the citizens’ satisfaction with the residential district by evaluating the environmental quality, infrastructure, and the feeling of safety in the place of residence, as well as the citizens’ concerns and possibilities of physical activity at the individual and the district levels in a large sample of subjects. The large sample size enabled us to investigate both main and potential confounding variables. Moreover, in this cross-sectional study, using stratified logistic regression models and controlling the studied associations for the possible confounding variables, we found that the recommended level of physical activity modified the effects of noise on hypertension in 45–64-year-old participants. This study presented evidence that the quality of the built environment and physical activity had a complex effect on the stress level and hypertension. The use of district-level neighborhoods for local environmental quality evaluation may increase the direct applicability of the study results in policy making.

However, our study also has several limitations. This is a cross-sectional study, and thus it does not provide causation as there is no direct evidence on the time sequence of the studied health effects. We only describe the existing associations between the environmental variables, physical activity, and health indices. Even though we controlled for possible confounding variables, residual confounding in self-reported personal characteristics and the reported physical activity level is possible. We acknowledge possible errors in self-reported evaluations of health problems as the result of recall bias associated with the participants’ age and social disparity. However, in this study, we included questions for checking the consistency in the participants’ answers on self-reported hypertension and physical activity. We found good concordance between the prevalence of self-reported physician-diagnosed hypertension and the representative sample of the inhabitants of Kaunas city [51]. Therefore, the results show a possibility for the generalization of the study findings to a large extent. In future studies, objective physical activity measurements using sensors would increase the societal awareness of the importance of physical activity for the prevention of chronic stress and hypertension.

## 5. Conclusions

This citizen science research created an opportunity for the participants to familiarize themselves with the local environmental problems, to engage in research planning, and to raise awareness about the links between environmental issues and health. The findings of this environmental epidemiological study are helpful in gaining a better understanding of the relationship between the quality of the neighborhood and individual-level characteristics, the level of the physical activity, and the magnitude of the prevalence of stress and hypertension. Our findings suggest that the quality of the built environment and the recommended level of physical activity had a complex effect on the stress level and hypertension and that the recommended physical activity level can modify the effects of noise on hypertension in 45–64-year-old participants. The implications of this research are that it suggests creating residential neighborhoods that would promote increased physical activity, such as reaching green spaces by walking and reducing noise level, which might reduce the risk of hypertension and improve well-being. Measures oriented towards healthy behavior should be encouraged among citizens in order to decrease the risk of chronic diseases.

## Figures and Tables

**Table 1:** Characteristics of the 18–75-year-old participants by physical activity level.

Characteristics	Total Number	PA < 150 min/Week, N (%)	PA ≥ 150 min/Week, N (%)	*p*
**Age groups**	1086			0.147 ‡
18–44	360 (33.1)	317 (88.1)	43 (11.9)	
45–64	668 (61.5)	558 (83.5)	110 (16.5)	
≥65	58 (5.3)	50 (86.2)	8 (13.8)	
**Sex**	1086			0.608 ‡
Men	498 (45.9)	421 (84.5)	77 (15.5)	
Women	588 (54.1)	504 (85.7)	84 (14.3)	
**District**	1086			0.114 ‡
1	84 (7.7)	68 (81.0)	16 (19.0)	
2	92 (8.5)	79 (85.9)	13 (14.1)	
3	124 (11.4)	106 (85.5)	18 (14.5)	
4	145 (13.4)	124 (85.5)	21 (14.5)	
5	84 (7.7)	73 (86.9)	11 (13.1)	
6	58 (5.3)	48 (82.8)	10 (17.2)	
7	72 (6.6)	53 (73.6)	19 (26.4)	
8	95 (8.7)	78 (73.6)	17 (17.9)	
9	118 (10.9)	106 (89.8)	12 (10.2)	
10	88 (8.1)	81 (92.0)	7 (8.0)	
11	126	109 (86.5)	17 (13.5)	
**Family status**	1086			0.796 ‡
Married	623 (57.4)	529 (84.9)	94 (15.1)	
Other	463 (42.6)	396 (85.5)	67 (14.5)	
**Education status**	1086			0.392 ‡
Lower educated	509 (46.9)	439 (86.2)	70 (13.8)	
Higher educated	577 (53.1)	486 (84.2)	91 (15.8)	
**Situation at work**	1084			1.000 ‡
Full-time	729 (67.3)	621 (85.2)	108 (14.8)	
Other	355 (32.7)	302 (85.1)	53 (14.9)	
**Monthly net income**	1086			0.815 ‡
<400 Euro	173 (15.9)	149 (86.1)	24 (13.9)	
≥400 Euro	913 (84.1)	776 (85.0)	137 (15.0)	
**Current smoking**	1082			0.280 ‡
No	810 (74.9)	695 (85.8)	115 (14.2)	
Yes	272 (25.1)	226 (83.1)	46 (16.9)	
**Traffic 10,000 cars/day**				0.219 ‡
<10,000	776 (71.5)	654 (84.3)	122 (15.7)	
≥10,000	310 (28.5)	271 (87.4)	39 (12.6)	
**Duration of living, years (mean (SE))**	17.61 (0.42)	17.76 (0.47)	16.67 (0.99)	0.361 †

† *p*-value of Student’s *t* test; ‡ *p*-value of the chi-squared test; SE—standard error.

**Table 2:** Self-reported health characteristics in 18–74-year-old participants’ groups by physical activity level.

Variables	PA < 150 min/Week, N (%) or Mean (SE)	PA ≥ 150 min/Week, N (%) or Mean (SE)	*p*
**18–44 years**			
Systolic blood pressure	121.59 (0.77)	127.21 (2.17)	0.010
Diastolic blood pressure	81.56 (0.68)	83.13 (1.73)	0.408
Hypertension			0.682
No	258 (81.4)	34 (79.1)	
Yes	59 (18.6)	9 (20.9)	
Stress level			0.870
Stress high (score < mean)	177 (55.8)	25 (58.1)	
Stress low (score > mean)	140 (44.2)	18 (41.9)	
**45–64 years**			
Systolic blood pressure	125.10 (0.58)	123.10 (1.36)	0174 †
Diastolic blood pressure	88.01 (1.07)	79.64 (2.97)	0.017 †
Hypertension			0.041 ‡
No	380 (68.1)	86 (78.2)	
Yes	178 (31.9)	24 (21.8)	
Stress level			0.007 ‡
Stress high (score < mean)	293 (52.5)	42 (38.2)	
Stress low (score > mean)	265 (47.5)	68 (61.8)	
**65–74 years**			
Systolic blood pressure	138.11 (2.37)	136.43 (10.16)	0.816
Diastolic blood pressure	87.16 (1.78)	80.57 (2.97)	0.165
Hypertension			
No	16 (32.0)	2 (25.0)	1.000
Yes	34 (68.0)	6 (65.)	
Stress level			0.697
Stress high (score < mean)	20 (40.0)	2 (25.0)	
Stress low (score > mean)	30 (60.0)	6 (75.0)	

† *p*-value of Student’s *t* test; ‡ *p*-value of the chi-squared test; SE—standard error.

**Table 3:** Mean ratings of the perceptions of neighborhood quality and social well-being in 45–64-year-old participants by the low and recommended physical activity level.

Questions Neighborhood Quality and Social Well-Being	PA Low Mean (SE)	PA Recommended Mean (SE)	*p*
The public transport in the district meets my needs	5.15 (0.080)	5.44 (0.161)	0.113
I am satisfied with pathways and cycling routes	4.85 (0.090)	5.20 (0.192)	0.111
There are opportunities for walking to reach the city’s green spaces or parks	5.26 (0.088)	5.67 (0.191)	0.054
I regularly visit the natural environment	4.66 (0.085)	5.46 (0.180)	0.001
There is a place in my residential area adapted for exercise and relaxation	4.41 (0.090)	4.50 (0.211)	0.695
The air pollution in my place of residence cause problems	3.98 (0.089)	4.33 (0.192)	0.116
The noise in my place of residence hinders my sleep and/or work at home	4.94 (0.084)	5.21 (0.183)	0.191
There are available public spaces and rooms to meet people in my residential area	3.99 (0.089)	4.49 (0.195)	0.025
I feel safe in my area	5.10 (0.073)	5.24 (0.161)	0.436
I can take part in decision-making to improve the environment in which I live	3.37 (0.091)	3.45 (0.203)	0.968
During the last 6 months, I have felt stress, tension, or anxiety	4.08 (0.079)	4.68 (0.164)	0.002

All neighborhood perception scores ranged from 1 to 7: 1 = strongly disagree, and 7 = strongly agree. Higher scores indicate better neighborhood conditions, less stress. PA, physical activity low <150 min/week, and PA recommended ≥150 min/week.

**Table 4:** Multivariable logistic regression models of neighborhood quality items on hypertension, diastolic blood pressure, and stress in 45–64-year-old participants.

Variables	Dependent Variable Models		
	Hypertension (Yes)	Diastolic Blood Pressure (≥90)	Stress (<Mean)
	Adjusted OR * (95% CI)	Adjusted OR * (95% CI)	Adjusted OR * (95% CI)
Quality of pathways and cycling routes			
<mean score	1.13 (0.80–1.61)	1.03 (0.67–1.57)	**1.80 (1.30–2.50)**
Green spaces by walking			
<mean score	1.21 (0.87–1.70)	1.25 (0.83–1.87)	**1.74 (1.27–2.38)**
Regular visits to green spaces			
<mean score	**1.80 (1.27–2.55)**	**2.09 (1.39–3.13)**	**1.46 (1.05–2.01)**
Available relaxation area			
<mean score	**1.69 (1.20–2.36)**	1.35 (0.90–2.02)	1.03 (0.75–1.40)
Air pollution problems			
<mean score	1.27 (0.91–1.78)	1.27 (0.85–1.90)	**1.68 (1.24–2.29)**
Noise problems			
<mean score	**1.47 (1.03–2.08)**	0.70 (0.45–1.09)	**1.81 (1.30–2.52)**
Safety in the district			
<mean score	0.82 (0.58–1.14)	0.81 (0.54–1.21)	**2.16 (1.58–2.95)**
Traffic 10,000 cars/day			
No	1	1	1
Yes	1.17 (0.81–1.69)	0.91 (0.57–1.43)	1.21 (0.86–1.71)
Physical activity			
Recommended	1	1	1
Low	**1.65 (1.01–2.67)**	1.67 (0.92–3.11)	**1.79 (1.18–2.72)**

OR, odds ratios; * adjusted for: sex, education level, age, smoking status, and income; Neighborhood quality scales ranged from 1 to 7. For all scales, the referent group is the mean score or above; Significant results are bolted.

**Table 5:** Multivariable logistic regression relationships between noise level, physical activity, and the risk of hypertension in 45–64-year-old participants.

Variables	N (%) Control	N (%) Cases	Crude OR (95% CI)	aOR ‡ (95% CI)
Low noise level (<mean) and recommended physical activity	64 (81.0)	15 (19.0)	1	1
High noise level (>mean) and recommended physical activity	22 (71.0)	9 (29.0)	1.75 (0.67–4.55)	1.93 (0.73–5.13)
Low noise level (<mean) and low physical activity	262 (70.6)	109 (29.4)	1.77 (0.97–3.25)	1.72 (0.93–3.18)
High noise level (>mean) and low physical activity	118 (63.1)	69 (36.9)	**2.49 (1.32–4.71)**	**2.25 (1.28–4.69)**

‡ Adjusted for: age, sex, education status, family status, and smoking status; The referent group is low noise level and recommended physical activity; Significant results are bolted.
